# What Contributes to the Measured Chiral Optical Response of the Glutathione-Protected Au_25_ Nanocluster?

**DOI:** 10.1021/acsnano.3c01309

**Published:** 2023-06-08

**Authors:** Marta Monti, María Francisca Matus, Sami Malola, Alessandro Fortunelli, Massimiliano Aschi, Mauro Stener, Hannu Häkkinen

**Affiliations:** † Dipartimento di Scienze Chimiche e Farmaceutiche, Università di Trieste, Via L. Giorgieri 1, 34127 Trieste, Italy; ‡ Department of Physics, Nanoscience Centre, University of Jyväskylä, P.O. Box 35, FI-40014 Jyväskylä, Finland; § CNR-ICCOM, Consiglio Nazionale delle Ricerche, via G. Moruzzi 1, 56124, Pisa, Italy; ⊥ Dipartimento di Scienze Fisiche e Chimiche, Università dell’Aquila, Via Vetoio, 67100 l’Aquila, Italy; ¶ Department of Chemistry, Nanoscience Centre, University of Jyväskylä, P.O. Box 35, FI-40014 Jyväskylä, Finland

**Keywords:** gold, nanocluster, thiols, chirality, molecular dynamics, density functional theory, essential dynamics

## Abstract

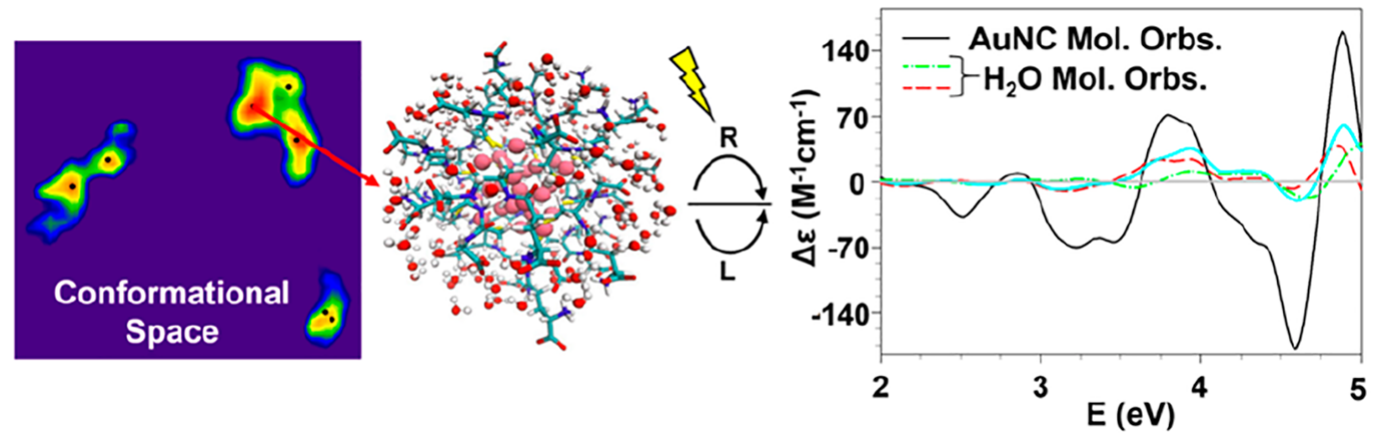

The water-soluble glutathione-protected [Au_25_(GSH)_18_]^−1^ nanocluster was investigated by integrating several methodologies such as molecular dynamics simulations, essential dynamics analysis, and state-of-the-art time-dependent density functional theory calculations. Fundamental aspects such as conformational, weak interactions and solvent effects, especially hydrogen-bonds, were included and found to play a fundamental role in assessing the optical response of this system. Our analysis demonstrated not only that the electronic circular dichroism is extremely sensitive to the solvent presence but also that the solvent itself plays an active role in the optical activity of such system, forming a chiral solvation shell around the cluster. Our work demonstrates a successful strategy to investigate in detail chiral interfaces between metal nanoclusters and their environments, applicable, e.g., to chiral electronic interactions between clusters and biomolecules.

Chirality in thiolate-protected gold nanoclusters (RS-AuNCs) was detected more than 20 years ago by Whetten and collaborators^1,2^ who worked on clusters with 20–40 Au atoms protected by the l-glutathione (GSH). Since then, significant progress has been made in the synthesis and total structural characterization as well as on the experimental and theoretical investigations of the electronic and chiroptical properties of RS-AuNCs.^3−14^ The current view suggests that the chirality in RS-AuNCs can be classified as intrinsic or induced. The intrinsic one arises when the metal core is chiral itself or can be related to chiral arrangements of the protective achiral ligands, while the second case regards chiral ligands which induce the chirality on the metal architecture. Moreover, several studies have been carried out investigating the peculiar optical properties of these atomically precise systems, which exhibit a discrete electronic structure when the size becomes smaller than *ca*. 2 nm.^15−18^ The interest in the discrete structures and properties of RS-AuNCs arises because of their potential applications in numerous fields such as catalysis,^19^ chemical sensing,^20^ optical devices,^21^ and biomedicine,^22^ providing possibility of tuning their properties according to size, shape, and composition.^23,24^

In this work, we focus on one of the first discovered RS-AuNCs, the chiral [Au_25_(GSH)_18_]^−1^, interesting especially in the biomedical field for the selective binding of target molecules such as the glutathione-*S*-transferase.^24^ While some initial confusion existed in the literature about its proper chemical composition,^1,25^ the chemical formula [Au_25_(SR)_18_]^±1^ was unambiguously identified based on several high-resolution mass spectroscopy experiments from 2005–2007.^26−29^ Over the last two decades, several efforts have also been made to understand the atomic structure of [Au_25_(GSH)_18_]^−1^ since its crystal structure is still unresolved. Indeed, despite the [Au_25_(GSH)_18_]^−1^ stability demonstrated by Shichibu and co-workers,^28^ the bulky and highly flexible GSH ligands make the crystal growth to high-quality single crystals very challenging. However, NMR studies by Wu and co-workers^30^ imply that the Au–S architecture is compatible with that found for [Au_25_(SCH_2_CH_2_Ph)_18_]^−1^^5,12^ hence with an icosahedral Au_13_ core and six Au_2_(GSH)_3_ motifs. Experimental circular dichroism (CD) spectra in the UV–vis region reported in more recent studies^30,31^ agree with the original data from Whetten and collaborators,^1^ reconfirming the assignment of the chemical composition.

Theoretical studies on the induced effects of chiral ligands on the electronic structure and chiroptical properties of GSH-protected small Au clusters are scarce. The only exceptions are a density functional theory study^32^ where cysteine was used as a small chiral model ligand for [Au_25_(SR)_18_]^−1^, excluding solvent effects, and studies of Au_18_(GSH)_14_^33^ and [Au_25_(GSH)_18_]^−1^^34^ using quantum mechanics/molecular mechanics (QM/MM) simulations, which showed that ligand dynamics and solvent effects are important for clusters’ structural and electronic properties. Although these results are in line with what is known for small molecules and biomolecules in various solvents,^35−38^ no attempts have been made up to date to understand the original CD data measured for [Au_25_(GSH)_18_]^−1^ over 20 years ago^2^ considering a full theoretical system modeling not only the relevant ligand but also dynamics (conformations) of the ligand shell and solvent–ligand interactions.

Here, we evaluate and analyze the UV–visible CD spectrum of [Au_25_(GSH)_18_]^−1^ by combining classical molecular dynamics simulations, essential dynamics analysis, and state-of-the-art time-dependent density functional theory calculations including conformational and solvent effects. We find that the aqueous solvent plays important direct and indirect roles in the chiroptical response of [Au_25_(GSH)_18_]^−1^ (see Figure 1) particularly in the UV energy range, where CD peaks can have up to 50% of the intensity originating from transitions involving electronic states of the solvation shell, which by itself has a chiral arrangement around the organic thiol surface. Our work demonstrates the importance to treat environmental factors properly in theoretical studies of RS-AuNCs in order to get a comprehensive understanding of their chiroptical response.

**Figure 1:**
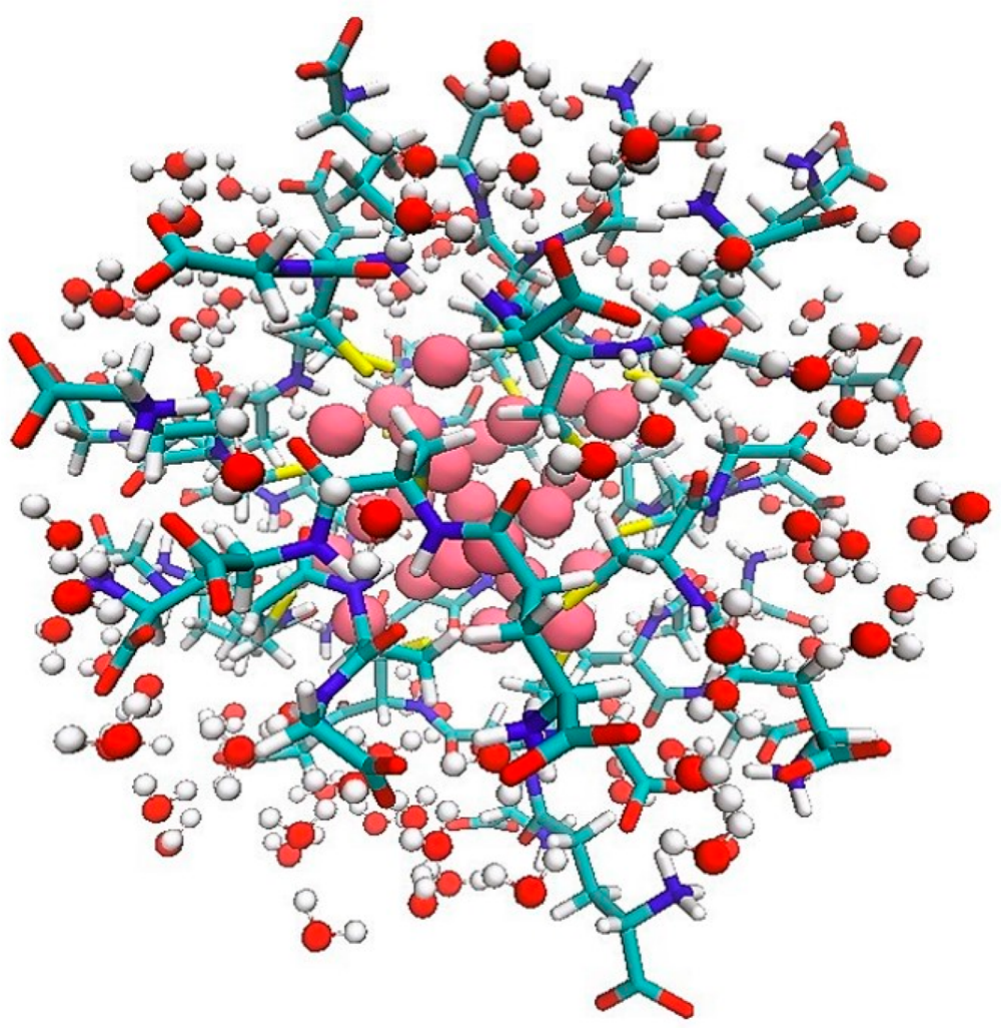
Stick and balls example of the [Au_25_(GSH)_18_]^−1^-(H_2_O)_126_ cluster conformation extracted with the procedure proposed. Au atoms are reported as pink balls, S, C, N, O, and H atoms of the GSH ligand are reported as sticks in yellow, cyan, blue, red, and white, respectively. A stick and ball model is used for the water molecules with O and H atoms in red and white, respectively.

## Results

### Full-Molecular Dynamics (MD) Simulation and [Au_25_(GSH)_18_]^−1^ Conformational States

**Figure 2:**
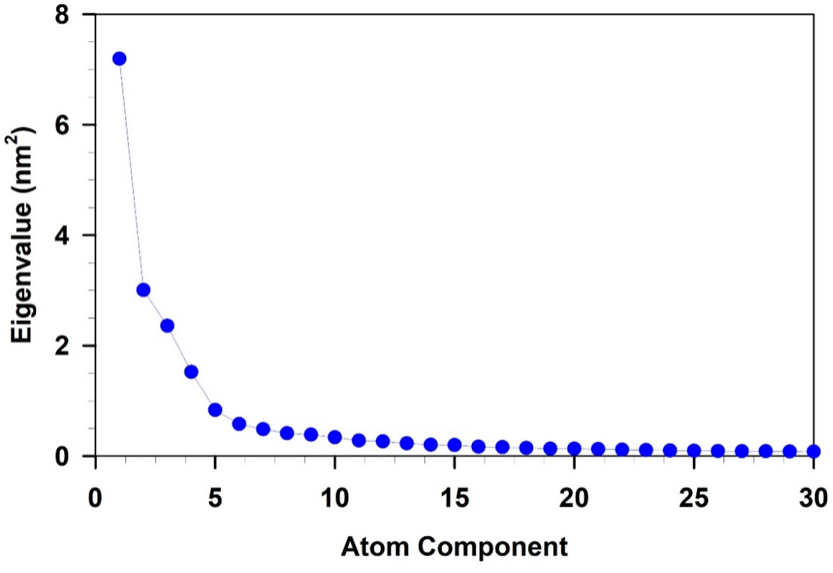
Eigenvalues of the [Au_25_(GSH)_18_]^−1^ covariance matrix at RT. Only the first 30 values are shown for the sake of clarity.

**Figure 3:**
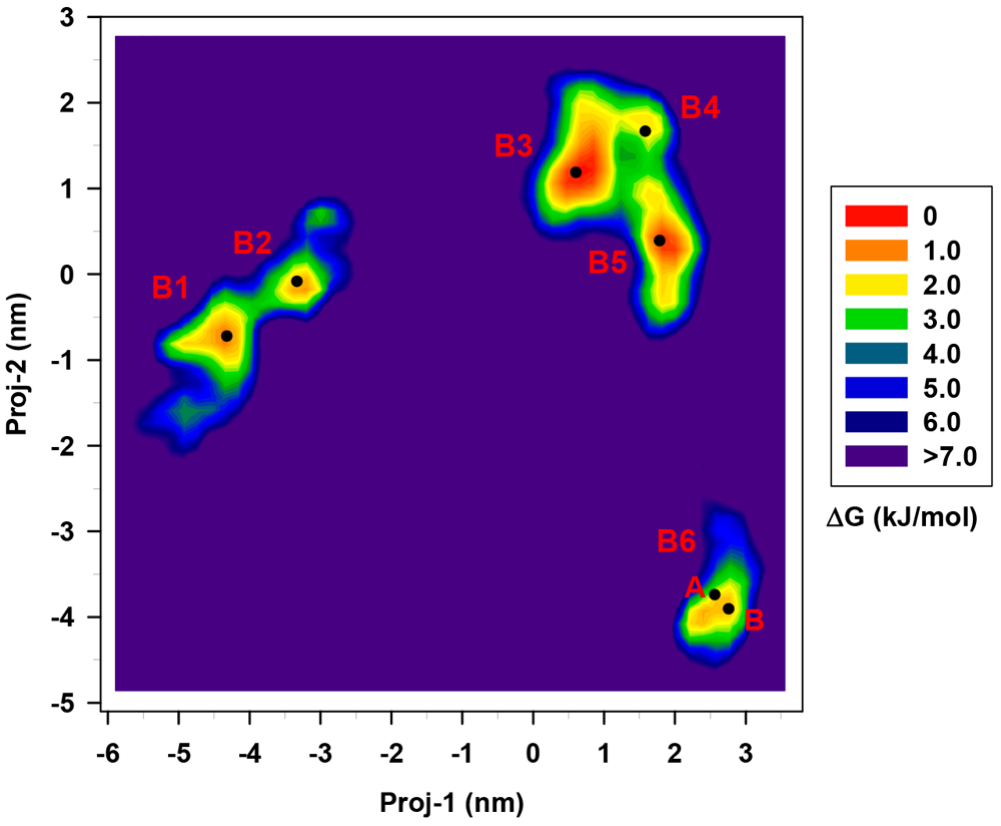
Free energy landscape in the essential plane of [Au_25_(GSH)_18_]^−1^ at RT. The energy scale in kJ/mol is reported as a vertical-colored bar on the right. All data with a Δ*G*° > 7.0 kJ/mol is shown by dark purple. The spatial positions of the 7 extracted most probable conformations are marked with black dots.

Figure 2 shows the spectrum of the first 30 eigenvalues extracted from the diagonalization of the nanocluster covariance matrix as obtained from the full-MD simulation. The overall picture shows that the eigenvalues, i.e., the mean square fluctuations of the system, rapidly decrease and a large fraction (around 60%) of the trace of the matrix (corresponding to the whole nanocluster fluctuation) arises from the first 5 eigenvalues. Such a relatively high number of essential eigenvectors, i.e., principal directions along which the internal motions happen, is clearly due to the size of the nanocluster, not only in terms of number of atoms but also in terms of internal flexibility. However, the first component accounts for 30% of this fraction, while another 12% results from the second component. Therefore, the further system’s conformational analysis was done considering only the corresponding first two eigenvectors to reach a computational affordable description of the physics of this system. Figure 3 shows the projection of the Cartesian coordinates of the nanocluster, at each frame of the MD trajectory, onto the first (proj-1) and second (proj-2) eigendirections of the covariance matrix shown in Figure 2.

The free-energy landscape reveals the presence of several shallow low-energy regions (with Δ*G*° < 2.5 kJ/mol), suggesting that along the full-MD simulation, the system is very flexible and lies in different stable, short-living, conformational basins. This result is expected considering the size, number, and flexibility of the GSH ligands, which can be involved in various conformational transitions. We can also notice that the low energy states in the conformational space are collected in three groups, with the interconversion being fast (barriers below 3 kJ/mol), between B1–B2, and B3–B4–B5, while being slow from one group to another. Such result corroborates again the complexity and high flexibility of the system which can rapidly interconvert from one stable conformation into another. We extracted one representative conformation per basin except for the region B6 where we found that the root–mean–square deviation (RMSD) of the two structures was higher than our threshold value of 0.17 nm. Energy details of the AuNC conformers are reported in Table S1 of the Supporting Information (SI).

**Figure 4:**
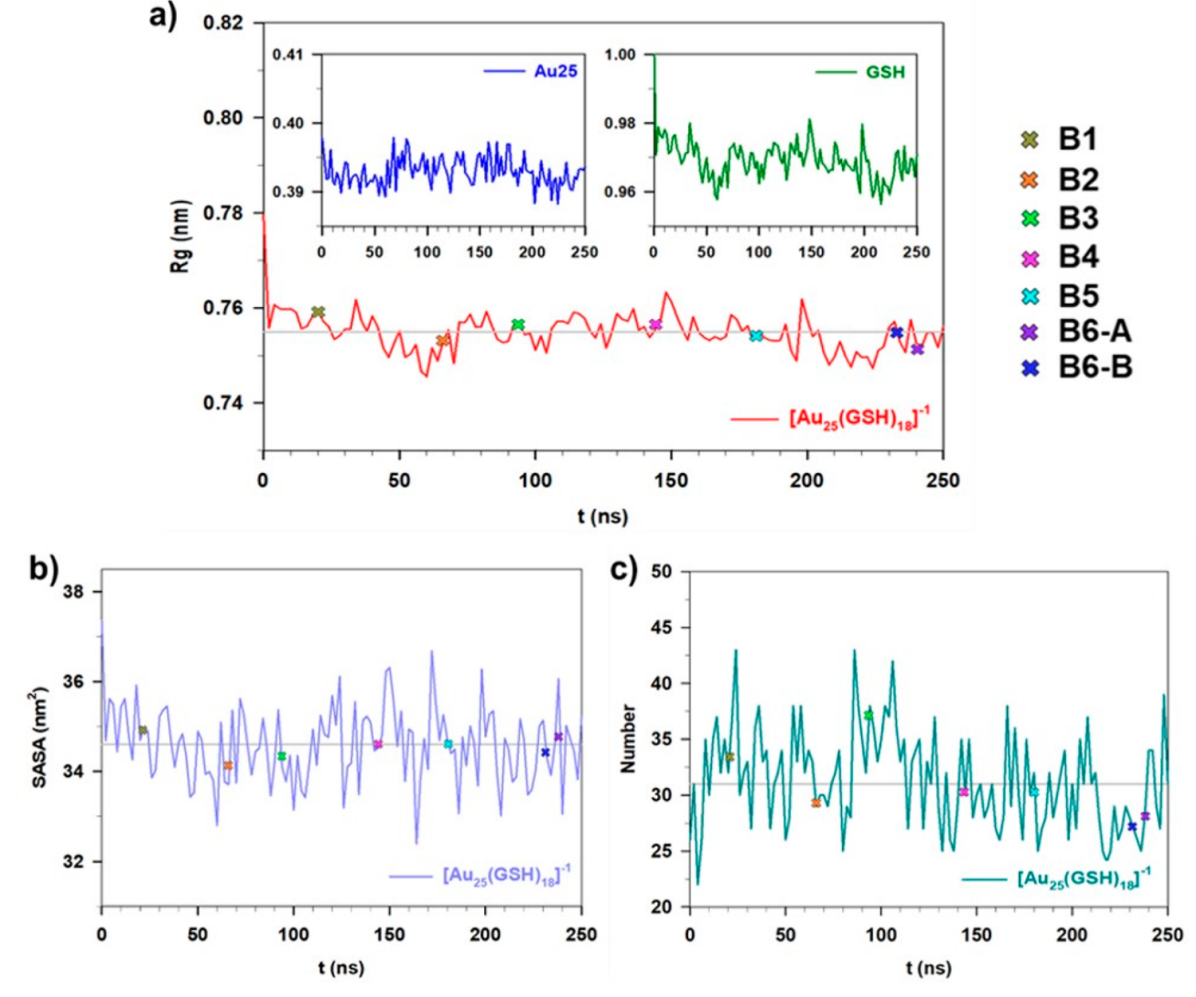
a) Radius of gyration (Rg, in nm), b) solvent accessible surface area (SASA, in nm^2^), and c) intramolecular H-bonds (a.u.) of [Au_25_(GSH)_18_]^−1^ (among the ligands) along the 250 ns full-MD simulation. In the upper panels of a), the Rg of Au_25_ and of the GSH ligands are reported as well. The 7 conformations selected with the previous ED analysis are reported with colored crosses (legend on the upper right region of the figure) in correspondence of their time step in the MD production.

Before proceeding with the investigation of the solvent shell, we extracted additional information on the selected conformers studying the radius of gyration (Rg), the solvent accessible surface area (SASA), and the intramolecular H-bonds of [Au_25_(GSH)_18_]^−1^ from the full-MD simulation. All the results are collected in Figure 4.

Fluctuations of the three quantities in Figure 4 (Rg, SASA, and intramolecular H bonds) are all relatively small, suggesting that our system is well equilibrated along the 250 ns MD simulation. This applies particularly to the radius of gyration (Figure 4a), which indicates the system compactness and the preservation of global shape along the MD. Therefore, the initial spherical shape of the nanocluster is conserved through the simulation, and the 7 stable conformations extracted do not display differences in terms of global compactness. A similar result is achieved for the surface area accessible to the solvent (see Figure 4b), where, again, for all the low-energy structures, we found a SASA value around the average one (i.e., 34.6 nm^2^).

Different results are obtained by analyzing the number of intramolecular H-bonds among the 18 GSH ligands of the nanocluster since some fluctuations are found for the 7 conformers. While for the B2, B4, and B5 conformations, the number is around the average value (i.e., 31), a higher number is observed for B1 and B3, in particular for the latter one (i.e., 37), which is also the most stable conformation. An opposite trend is obtained for the structures selected from the B6 region (see Figure 3), where the number of H-bonds is reduced (i.e., 27/28). It is well-known that the presence of H-bonds affects the optical response of the system,^37,38^ especially for the highly sensitive ECD technique. Therefore, we expect the intramolecular H-bonds, together with the intermolecular ones (see Figure S1 for a detailed analysis), to have a clear impact on the ECD spectrum.

### Constrained-MD Simulations and [Au_25_(GSH)_18_]^−1^-(H_2_O)_126_ Clusters Conformational States

**Figure 5:**
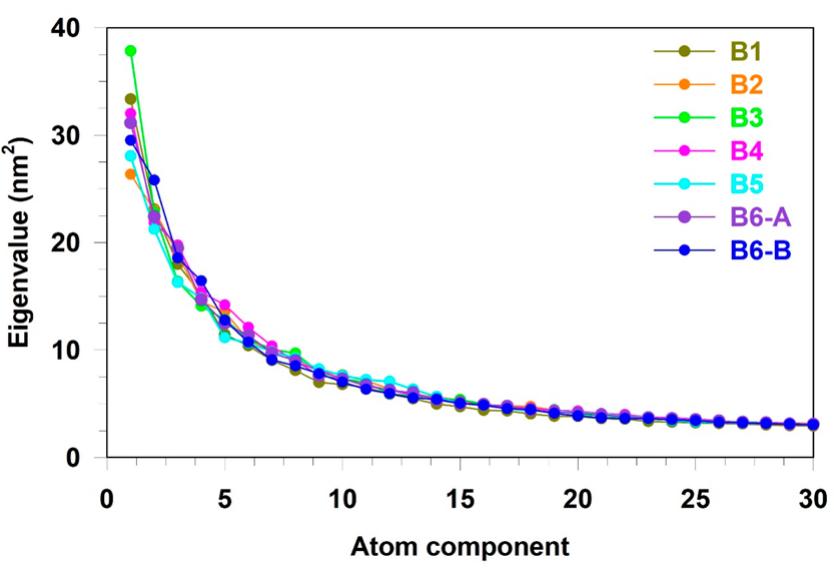
Eigenvalues of the [Au_25_(GSH)_18_]^−1^-(H_2_O)_126_ clusters covariance matrix obtained from the constrained-MD simulations at RT. Only the first 30 values are shown for the sake of clarity.

**Figure 6:**
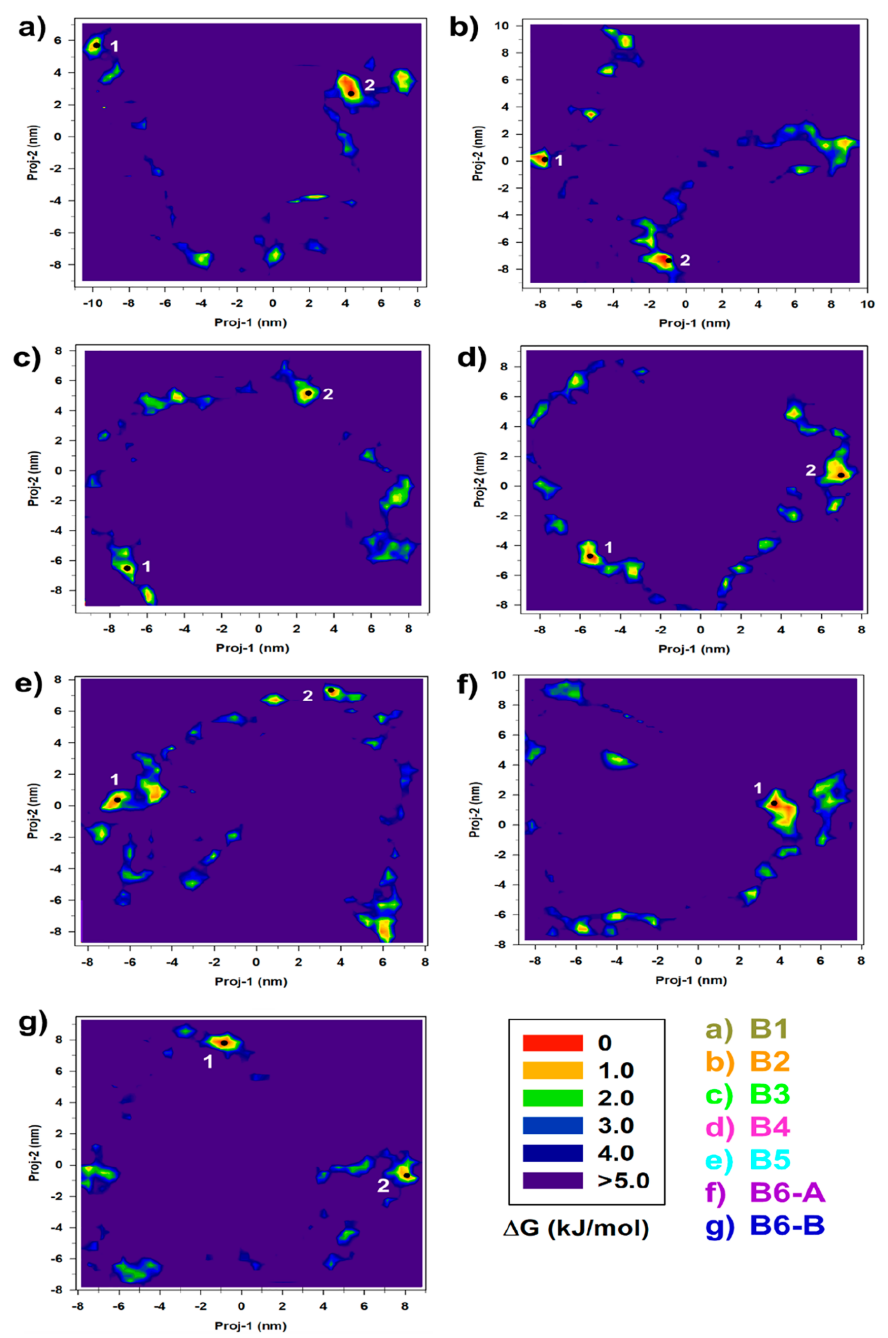
Free energy landscapes in the essential planes of the [Au_25_(GSH)_18_]^−1^-(H_2_O)_126_ clusters from the constrained-MD simulations at RT. The energy scale in kJ/mol is reported as a vertical-colored bar on the lower right-side of the figure together with the labels of the [Au_25_(GSH)_18_]^−1^ conformations used in each constrained-MD (and extracted from the previous full-MD). All data with a Δ*G*° > 5.0 kJ/mol are shown by dark purple. The spatial positions of the selected most probable cluster conformations are marked with black dots.

The characterization of the solvation shell is, in general, a nontrivial task because of the need to find the actual number of solvent molecules defining the different solvation shells (see the Computational Details and Figures S3 and S4 of the SI) and, most importantly, because of their relatively high mobility. In this study, we have adopted a strategy based on ED which, similarly to what has been described in the previous section, allows us to span the conformational repertoire of a preselected number (126) of solvent molecules with respect to the (frozen) [Au_25_(GSH)_18_]^−1^ conformations extracted from Figure 3. The analysis is in principle rigorous, but very difficult in practice, because of the high mobility of the solvent molecules. As a matter of the fact, the spectra of the first 30 eigenvalues reported in Figure 5 show this significant increase in the overall fluctuation (the trace of the covariance matrix) and its spread over a high number of internal degrees of freedom. Despite the reduced weight of the first two eigenvalues, thus some possible limitations, we proceeded with a 2D conformational investigation for computational reasons. The resulting free-energy landscapes, one for each of the 7 [Au_25_(GSH)_18_]^−1^ conformations, reported in Figure 6 show a series of important features which deserve some comments.

First, if compared to the analogue conformational space reported in Figure 3, we observe that, as expected, the [Au_25_(GSH)_18_]^−1^-(H_2_O)_126_ system spans a space much larger than the [Au_25_(GSH)_18_]^−1^ one, also showing a higher number of conformational states. These findings obviously reflect the larger number of internal degrees of freedom and the higher mobility of the aqueous solvent. In addition to that, the spots locating the stable [Au_25_(GSH)_18_]^−1^-(H_2_O)_126_ appear as very separated onto the conformational space, thus suggesting the presence of high interconversion barriers. This result is the effect of both the use of a reduced 2D projection subspace and a reflection of the complex interaction between the nanocluster and the solvent. Indeed, several kinds of weak interactions (charge–dipole, dipole–dipole, H-bonds) can define the system, with the water molecules found both in the inner and outer region of [Au_25_(GSH)_18_]^−1^. The spatial positions of the selected most probable cluster conformations are marked with black dots.

We then decided to extract one or two conformations among the most probable ones (with *ΔG*__*H*2*O*_^°^_ < 1.0 kJ/mol) from almost opposite regions of each landscape to maximally increase the heterogeneity of our reduced final set of structures. Details of the 13 [Au_25_(GSH)_18_]^−1^-(H_2_O)_126_ clusters conformations, which have been subsequently used for the calculation of the UV-CD spectra, are collected in Table 1.

**Table 1:** Δ*G*° and Normalized Probability Values of the 13 [Au_25_(GSH)_18_]^−1^-(H_2_O)_126_ Conformations Extracted from the ED Analysis of Constrained-MD Simulations

[Au_25_GSH)_18_]^−1^-(H_2_O)_126_ Conformation	Δ*G*_TOT_^°^ (kJ/mol)^a^	p(j,i)_norm_^b^
B1–1	1.61	0.067
B1–2	0.87	0.090
B2–1	2.05	0.057
B2–2	1.45	0.072
B3–1	0.00	0.13
B3–2	0.98	0.087
B4–1	2.21	0.053
B4–2	2.82	0.041
B5–1	0.81	0.093
B5–2	0.34	0.11
B6-A–1	2.03	0.057
B6–B–1	1.40	0.073
B6–B–2	1.51	0.070

a Δ*G*_TOT_^°^ = Δ*G*__[Au25GSH18]–1_^°^_ + Δ*G*__H2O_^°^_.

b ∑p(j,i)_norm_ = 1.

### UV-Circular Dichroism (UV-CD) Spectra

**Figure 7:**
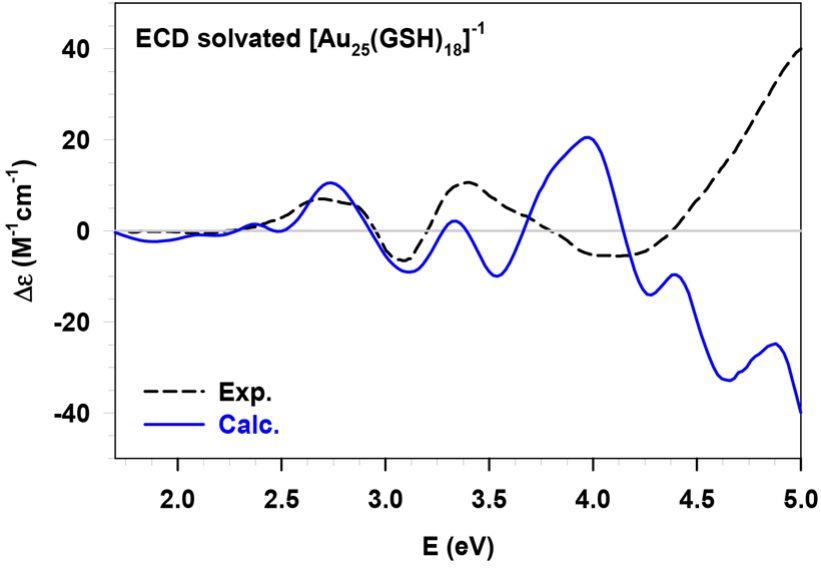
Experimental (Exp., black dashed line, ref (2)) and calculated (Calc., solid blue line) ECD spectra of the solvated [Au_25_(GSH)_18_]^−1^ nanocluster. The calculated spectrum has been obtained by statistically weighting the 13 [Au_25_(GSH)_18_]^−1^-(H_2_O)_126_ cluster conformations extracted with the ED analysis.

The 13 individual UV-CD spectra were calculated, statistically weighted, and summed up to give the final averaged UV-CD spectrum, which was then compared with the experimental data available in the literature^2^ and reported in Figure 7.

Figure 7 highlights discrepancies and agreements between the experimental and calculated spectra. In the low energy region, where the signal arises from the ECD of the metal cluster, we observed an excellent agreement in terms of energy and intensity. As discussed in previous studies on this system,^30,34^ the achiral metal core shows an optical activity induced by the GSH ligands that disrupt the symmetry. Therefore, the employment of an MD simulation becomes fundamental to capture the metal structure distortion driven by the ligand fluctuations and interactions. In general, the calculated profile matches properly the experimental data up to 3.3 eV; beyond this value, the agreement looks less satisfactory. Indeed, from 4.3 eV onward, the discrepancies between the two spectra become more pronounced. Figure 7 shows that while the experiment presents a maximum positive peak toward 5 eV, the opposite phase is obtained for the calculated ECD spectrum.

In this high energy region, the ECD is more influenced by the solvent, which affects both the geometry and the electronic structure of the chiral species.^38^ Hence, some discrepancies can be justified considering that the water effects have been partially included in our work (limited solvation shell) because of the computational cost. However, by investigating the water role with different analyses (see subsection), we were able to point out in detail its strong effect on the AuNC chiral activity, thus confirming the need of its explicit contribution in the ECD calculations.

**Figure 8:**
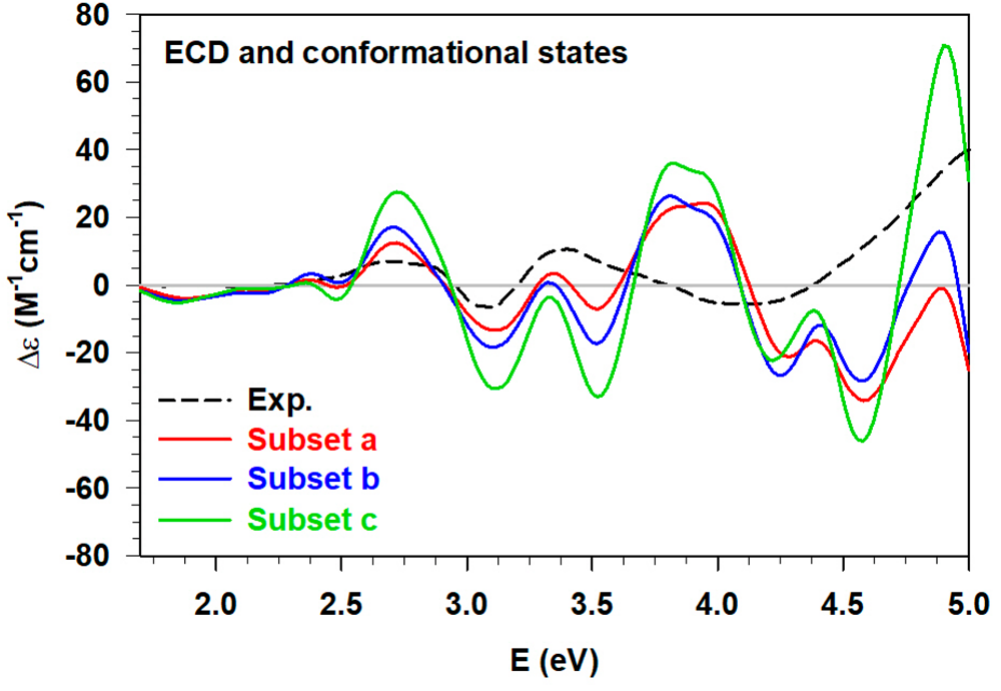
Experimental (Exp., black dashed line, ref (2)) ECD of the solvated [Au_25_(GSH)_18_]^−1^ in comparison with the calculated statistical ECD obtained considering only the most probable [Au_25_(GSH)_18_]^−1^-(H_2_O)_126_ conformations from 1) each conformational landscape (subset a, solid red line), 2) each conformational landscape except for the two B6 spaces (subset b, solid blue line), 3) B3, B4, and B5 conformational landscapes (subset c, solid green line).

Some limitations of our ECD spectrum can be also related to an imbalance of the conformational states, thus meaning that some of the structures we extracted are less probable in the experimental sample. This can be ascribed to limitations of the classical MD simulations which result in the inability to reproduce all the thermodynamics properties of the AuNC, thus producing artifacts in the ED analysis. However, upon analyzing the individual ECD spectra (see Figure S1), we noticed that the most probable conformer of each solvation free-energy landscape (Figure 6) shows spectral features in closer agreement with respect to the experimental ones. Moreover, the conformers extracted from the B6 region deviate from the general trends (e.g., H-bonds analysis, see Figures 4c and S1) followed by the other stable structures. Starting from these observations, we have built subsets of the original sample of conformers and renormalized their probabilities. Practically, we have considered: a) only the most probable conformer from each [Au_25_(GSH)_18_]^−1^-(H_2_O)_126_ free-energy landscape, and b) the same conformers but excluding the B6 regions. For investigating these hypothetical limitations of the MD simulations, we also built a subset c where we focus only on the most probable region of the conformational landscape (basins B3, B4, and B5 in Figure 3) which should be the most reliable of our sampling. All the new probability values reported in Table 2 were used to statistically weight the individual ECD spectra. The three averaged ECD are shown in Figure 8 together with the experimental spectrum.

**Table 2:** Renormalized Probabilities Considering Only the Most Probable Conformation from a) Each [Au_25_(GSH)_18_]^−1^-(H_2_O)_126_ Conformational Landscape, b) All the Landscapes except for the B6 Regions, c) Only B3, B4, and B5 [Au_25_(GSH)_18_]^−1^-(H_2_O)_126_ Conformational Spaces

[Au_25_GSH)_18_]^−1^-(H_2_O)_126_ Conformation	p(j,i)_norm_, Subset a	p(j,i)_norm_, Subset b	p(j,i)_norm_, Subset c
B1–2	0.16	0.20	//
B2–2	0.12	0.16	//
B3–1	0.22	0.28	0.44
B4–1	0.090	0.12	0.18
B5–2	0.19	0.25	0.38
B6-A–1	0.097	//	//
B6–B–1	0.13	//	//

It is worthy of note that considering a reduced sample of conformations (i.e., subset c), we obtained the correct intensity ratio between the high-energy ECD peaks and a very good agreement with the experimental spectrum. Indeed, while the spectral features of the metal region remain almost unchanged in all the cases, except for the intensity, significant differences are found for the two peaks located around 3.8 and 4.9 eV, respectively. Despite the blueshift of the *middle* maximum value, there is an improvement removing the B6 conformers (subsets b, and c), with the calculated maximum peak and its shoulder both correctly reproduced. Furthermore, if we consider only the most probable conformers from B3, B4, and B5 (subset c), the metal feature and the high energy maximum peak are in a very good agreement with the experimental ones in terms of energy position and intensity. These results corroborate the hypothesis that along both the full- and constrained-MD simulations there are some imbalances in the statistical weights and certain regions we investigated should be associated to higher free-energy values. However, by adjusting some details of the statistical analysis, we obtained a qualitative agreement between the experimental and calculated ECD, reproducing all the relevant spectral features.

### Water Effect on the Chiroptical Properties of [Au_25_(GSH)_18_]^−1^

**Figure 9:**
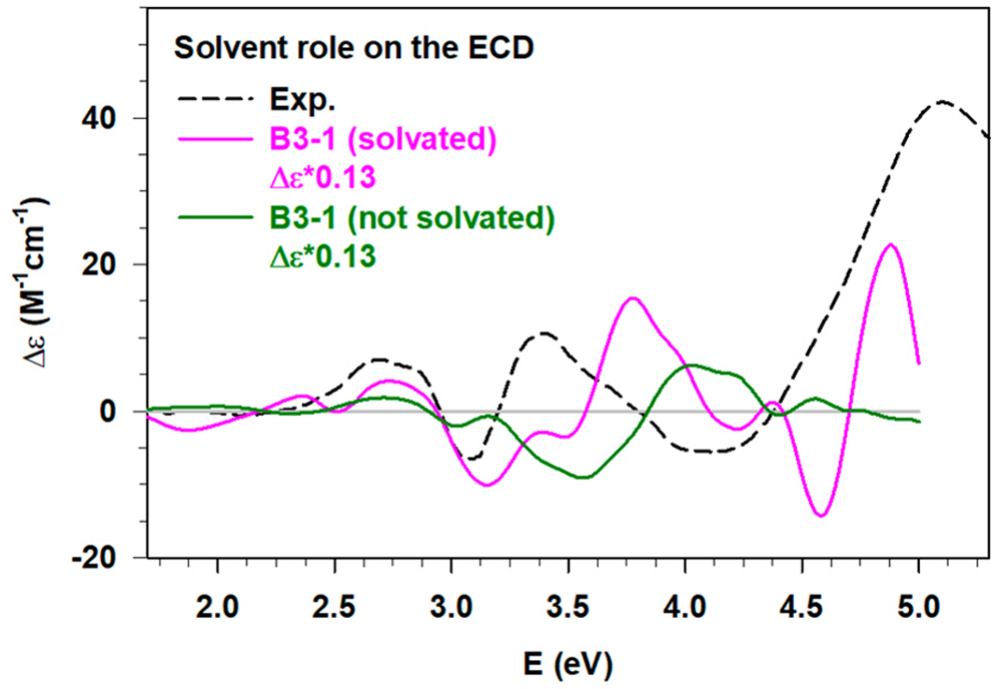
Experimental (Exp., black dashed line, ref (2)) ECD in comparison with the ECD calculated for the most probable conformation (B3–1) considering the solvation shell (solid pink line) and removing the water molecules (solid dark green line). The calculated spectra have been multiplied by the statistical weight of B3–1 (0.13) obtained from the ED analysis (see Table 1).

The role of water on the RS-AuNC chirality is clearly shown below comparing the ECD spectra of B3–1, the most probable conformer, in presence and absence of the solvation shell (Figure 9).

Indeed, while the ECD of the solvated B3–1 conformation in Figure 9 (pink line) reproduces quite well the experimental spectrum, several discrepancies are observed when the water molecules are removed (dark green line). Such differences affect even the inner metal region of the AuNC which does not directly interact with the solvent, for instance causing a reduction of intensity of the peak around 2.7 eV but also considering the features below 2.5 eV. In the region between 3 and 4 eV, we find that the experimental positive feature is reproduced much better in the solvated system. The discrepancy becomes much more evident considering the 4–5 eV energy range where the experimental positive peak is reproduced only in the solvated system. These results clearly point out the effect of the polar solvent on the RS-AuNC chiral response, thus the unambiguous need of including, at least, a partial explicit aqueous shell. It should be noted that in Figure 9 the calculated profiles are more structured than the experimental spectrum. This is most likely an effect of our limited sampling of cluster fluctuations corresponding to the experimental temperature. It is known that the experimental absorption spectra are quite sensitive to the temperature.^18^

**Figure 10:**
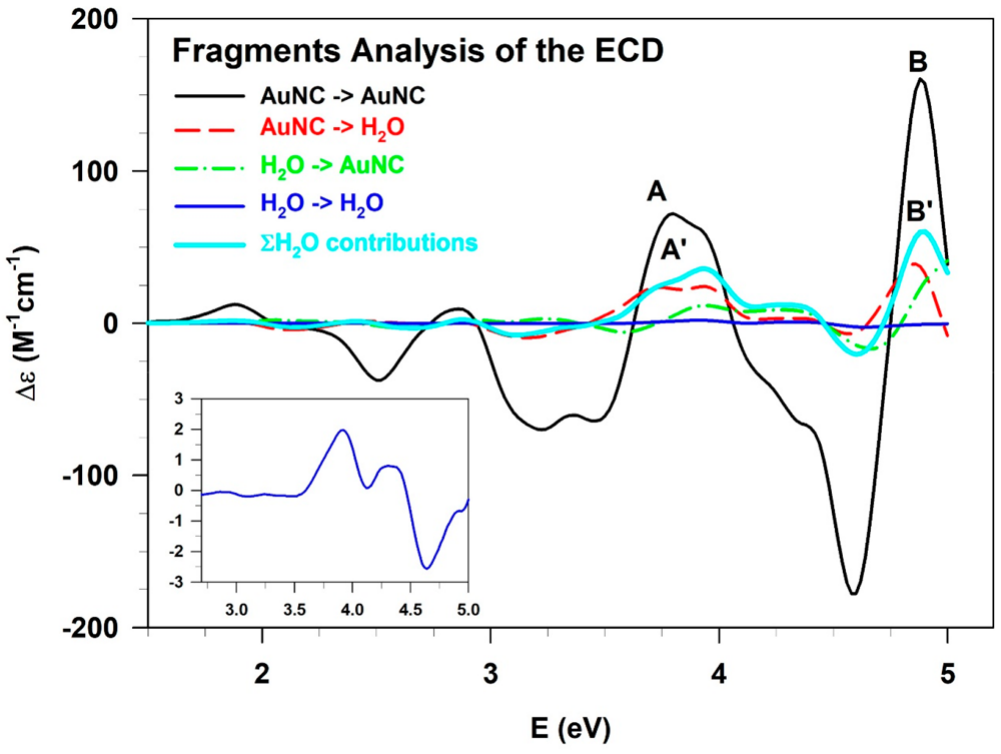
Fragment analysis of the ECD spectrum calculated for the most probable conformer, B3–1. The contributions of the AuNC and H_2_O molecular orbitals (initial MOs → final MOs) to the chiroptical properties are pointed out using different colored lines. The H_2_O → H_2_O transitions are highlighted in the bottom left inset of the figure.

The role of water has been also assessed by splitting the contributions of the AuNC (fragment 1) and the solvation shell (fragment 2) molecular orbitals (MOs) to the spectral features. Practically, employing a fragment analysis postprocessing tool available in AMS,^39,40^ it is possible to understand which MOs are involved in the transitions of the ECD spectrum of the whole system (B3–1 conformer), as shown in Figure 10.

It is worth noting that while in the low energy range the ECD response arises from transitions involving only the AuNC MOs, as expected, the two positive features characterizing the spectrum from *ca*. 3.5 eV onward show significant contributions of the water orbitals. More in detail, the intensity ratios between peaks A/A′ and B/B′ are about 0.5 and 0.4, respectively, thus highlighting the non-negligible contribution of the solvent to the ECD. Furthermore, in the same energy range, we can also observe H_2_O → H_2_O transitions, although their intensities are much lower compared to those involving the AuNC MOs. This result suggests that the solvation shell itself assumes a chiral arrangement interacting with the nanocluster. The role of the intermolecular interactions (e.g., H-bonds) in relation with the ECD response has been also extensively discussed in the SI. In addition to that, the ECD spectrum of only the water shell of the B3–1 conformation has been calculated up to 10 eV, corroborating again the chiroptical features of the solvent itself, particularly strong in the vacuum-UV energy range (see Figure S2). The chirality of [Au_25_(GSH)_18_]^−1^, the (H_2_O)_126_ shell, and the whole B3–1 system has been also evaluated in terms of Hausdorff chirality measure^41,42^ (HCM, see Table S2), obtaining a significant HCM value for both the components of the system.

The solvent effects on the ECD have been already discussed in the literature, as Giovannini et al.^35^ highlighted the effects of “chirality transfer” as well as the importance of the formation of hydrogen bonds. Chiroptical properties in solution have been studied also by Del Galdo,^36^ while Mancini^43^ developed an automated procedure based on evolutionary algorithm.

Therefore, the water shows several effects on the chiroptical properties of RS-AuNCs, such as polarization effects which affect the energy, and intensity of the nanocluster ECD features, but also a direct and significant contribution to the spectral transitions. Furthermore, the achiral solvent shows a low intensity ECD response itself due to the AuNC-H_2_O interactions. All these results prove the undeniable need to include explicitly the water molecules for a proper calculation of the RS-AuNCs chiroptical features.

## Conclusions

We have investigated in detail all factors that contribute to the measured ECD spectrum of the chiral, water-soluble [Au_25_(GSH)_18_]^−1^ nanocluster combining molecular dynamics simulations, essential dynamics analysis, and state-of-art time-dependent density functional theory calculations. Fundamental aspects such as conformational, weak interactions, and solvent effects have been considered in our approach since they strongly define the physics of this system. Our work showed that the glutathione-protected Au_25_ cluster probes several local basins of different ligand conformations during the molecular dynamics simulations, and statistical sampling of the dynamics becomes an important issue. We also revealed a clear effect of the aqueous environment on the chiroptical properties of RS-AuNCs, thus the need to treat explicitly the solvent in these calculations. In order to trace out a logical analysis of the relationship between the solvent role and the ECD, we compared the calculated ECD (averaged on the conformations) with the available experimental data, which are in good agreement in the low energy interval (where excitations are dominated by orbitals mostly centered on metal atoms) but show more important deviations in the high energy interval (whose excitations are dominated by orbitals mostly centered on the ligands). In order to understand the origin of such deviation in the high-energy region, we restricted the average only to the most probable conformers, obtaining a definite improvement on the overall agreement between theory and experiment. This clearly indicates that the origin of the disagreement between theory and experiment is due to an imbalanced treatment of the conformational degrees of freedom, and that restricting sampling to the most probable conformers leads to a good agreement with experiment; in particular, it is able to reproduce correctly the ECD maximum at 5 eV. Therefore, we have used this approach of considering only the most probable conformer to focus on the role of the solvent, and compared the spectrum with or without the presence of the solvent explicit molecules. We have thus been able to show that the ECD peak at 5 eV disappears if the solvent is not included, thus unequivocally demonstrating the active role of water molecules to assess the spectral features of the ECD. Analyzing the molecular orbital contributions to the ECD transitions, we were able to corroborate the direct role on the chiroptical features of the solvation shell, which also assumes a chiral arrangement itself around the organic thiol surface. Our work demonstrates a successful strategy to investigate in detail chiral interfaces between metal nanoclusters and their environments, applicable, e.g., to interactions between clusters and biomolecules for chiral sensing.

## Methods

### Molecular Dynamics Simulations

The coordinates of [Au_25_(GSH)_18_]^−1^ nanocluster were taken from ref (34). MD simulations were carried out with GROMACS software version 2021.4.^44^ using a previously published AMBER-compatible force field for nanocluster’s Au–S interface^45^ and standard parameters^46^ for the GSH ligands. The nanocluster was simulated in a cubic box containing TIP3P water^47^ and sodium ions to neutralize the system. The volume of the box and the number of solvent molecules were chosen in order to reproduce the concentration used in the experimental reference (i.e., 20 mg/mL).^2^ Energy minimizations were performed using the steepest descent algorithm, followed by a 10 ns equilibration in the NVT ensemble at 300 K and 10 ns equilibration in the NPT ensemble at 1 bar. Along these MD simulations, we constrained the internal degrees of freedom of the water molecules with the SETTLE algorithm^48^ as well as the position of the heavy atoms of the nanocluster.

Afterward, the position restraints were removed, and a production MD of 250 ns was performed in the NVT ensemble using the velocity-rescale thermostat.^49^ The Particle Mesh Ewald method^50^ with a cutoff of 1.0 and 0.12 nm grid spacing to treat the long-range electrostatic interactions was used. All the bonds were constrained with the LINCS algorithm^51^ to improve the performance. These simulations (here termed full-MD) were subsequently analyzed using the ED and followed by additional MD simulations of 25 ns where we kept frozen the nanocluster in each of the previously extracted conformations. These constrained-MD simulations were performed using the same conditions reported above for the full-MD and analyzed with the ED to map the conformational landscape of the solvation shell closer to each selected nanocluster conformation. Both ED analyses are described in the following section.

### Essential Dynamics (ED) Analysis

The features of the ED are widely discussed in the literature^52,53^ and the procedure used in this work has been described in detail in ref (38). Therefore, we summarize herein only the relevant aspects of the performed analysis. For the full-MD, we considered the first pair of eigenvectors, obtained by diagonalizing the covariance matrix of the [Au_25_(GSH)_18_]^−1^ atomic coordinates, which correspond to the two highest eigenvalues (mean square fluctuations) accounting in this way for most of the nanocluster conformational transitions. In principle, we could consider more than 2 eigendirections,^54^ at least the third one, thus conducting the conformational analysis in a *N* > 2 hyperspace. However, such analysis is very demanding and sometimes unnecessary for a still good description of the conformational transitions.^38,55^ Therefore, we projected the trajectory along these eigenvectors to obtain the principal components which are the starting point to build a 2D histogram, i.e., to obtain a representation of the 2D conformational landscape of the nanocluster. Each region of this space represents a conformational basin (i) whose probability (P(i)) depends on how frequent is that conformation. Starting from P(i) and P_ref_, which is the probability of the most probable basin, and assuming as negligible the difference between the volumes (NVT ensemble), we can calculate the standard Gibbs free energy difference (Δ*G*°) between these basins by using the Boltzmann statistics:
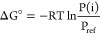
1

The calculation is repeated for each conformational region obtaining the free-energy landscape to investigate. As a result of this analysis, we extracted 7 conformers among the most probable ones (0 < Δ*G*° < 2.5 kJ/mol) sampling the different low-energy regions of the landscape (see Figure 3) and assuming as identical the conformations with a RMSD < 1.70 Å.^55^

We repeated the ED analysis on the constrained-MD simulations searching for plausible nanoparticle–solvent clusters to be used for the following quantum-chemical calculations. Details on how to perform this analysis are reported in the literature^38,56^ and here only summarized. We started constructing the fixed ellipsoid that best describes the nanocluster shape for each of the 7 constrained-MD, followed by the extraction of the N water molecules that show the lower square distances in the just defined ellipsoidal metrics. This step was repeated for each frame of the constrained-MD, obtaining a trajectory of the [Au_25_(GSH)_18_]^−1^-(H_2_O)_N_ cluster. We selected 126 solvent molecules balancing the number of sites per ligand that can interact directly with the water molecules (i.e., ∼9 sites) and those involved in intramolecular interactions (i.e., ∼2 sites). The latter parameter was estimated considering the average number of intramolecular H-bonds along the full-MD (see Figure 4c). Additional details on the selected water shell are shown in the SI (see Figure S3 and S4). This solvation, even though reduced, allows a compromise between the computational cost of the subsequently quantum-chemical calculations and the accuracy in representing the physics of the real aqueous system. We performed the ED analysis on the 7 obtained [Au_25_(GSH)_18_]^−1^-(H_2_O)_126_ clusters to study their conformational landscapes, i.e., the solvation shell conformations. From each space, we extracted one or two cluster conformations associated to a probability p(j,i) and a relative free energy Δ*G*° < 1.0 kJ/mol calculated by using eq 1. We obtained a final set of 13 [Au_25_(GSH)_18_]^−1^-(H_2_O)_126_ cluster conformations (see Figure 1 for an example) with their statistical weights, then used for the quantum-chemical calculations.

### Quantum-Chemical Calculations

The geometries of the 13 different conformations of the [Au_25_(GSH)_18_]^−1^-(H_2_O)_126_ clusters were optimized at the DFT level^57^ by using the Amsterdam Density Functional (ADF) engine of the AMS code.^58^ All the optimizations were performed employing the GGA Perdew-Burke-Erzerhof (PBE) exchange-correlation (xc) functional^59^ in combination with the GRIMME-D3 dispersion terms,^60^ an electron smearing of 0.05 Hartree to help the self-consistent field (SCF) convergence, a basis set of Slater-type orbitals (STO) of triple-ζ plus polarization quality (TZP), and the Zero Order Regular Approximation (ZORA)^61^ for the scalar relativistic effects. Default optimization geometry convergence criteria have been adopted (10^–5^ Hartree for energy, 0.001 Hartree/Å for nuclear gradients and 0.01 Å for the Cartesian step).

The ECD calculations were carried out with the complex polarizability TDDFT (pol-TDDFT) algorithm,^62^ also available in the AMS software.^58^ The algorithm defines the rotatory strength as
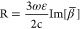
2where Im[β̅] represents the imaginary part of the rotatory strength tensor averaged over all the orientations, ω is the photon energy, c is the speed of the light, and ε is the imaginary part of the photon energy, here taken equals to 0.15 eV. Since the pol-TDDFT calculations are intrinsically broadened by a Lorentzian function, we can directly compare our results with spectra having the half-width half-maximum (HWHM) equal to ε. The ECD calculations were done with the asymptotically corrected LB94 xc functional,^63^ the TZP basis-set (appropriately optimized to be used with the pol-TDDFT),^64^ and the scalar ZORA approach.^61^ We neglected the spin–orbit coupling, since it is important only when isolated transitions are present, like in the lowest part of the spectrum.^65^ However, when many transitions are close to each other in energy, like in the present system, the broadening of the spectrum tends to wash out the effects of the spin–orbit coupling.^66^ For the most probable conformer, we also performed an ECD calculation with the GGA PBE, comparing the results with both the xc functionals with respect to the experimental spectrum (see Figure S5). So far, we have not tested hybrid functionals (e.g., B3LYP) since they can be included in a pol-TDDFT calculation only employing the hybrid diagonalization approximation (HDA),^67^ which is prohibitively expensive for such a large system. Furthermore, for the same most probable structure, we calculated the ECD only on the nanocluster conformation, thus removing the solvent molecules, to evaluate the effect of the explicit inclusion of the solvation shell on the optical response. The solvent impact on this [Au_25_(GSH)_18_]^−1^ nanocluster has been already discussed from a different perspective in Rojas-Cervellera et al.’s work^34^ and here evaluated in relation with the ECD response (see subsection). The role of the solvent has been also corroborated by performing the fragment analysis available as a postprocessing tool in AMS.^39,40^ Considering the NC and the solvation shell as different fragments, it is possible to split the two fragment contributions to the chiro-optical features, thus pointing out if the water orbitals contribute directly to the ECD transitions.

The ECD spectra of all the conformations were finally weighted with their relative p(j,i) values and summed to give the statistically averaged ECD. The overall calculated spectrum was compared with the experimental ECD data^2^ to evaluate the quality of the proposed approach.

## Supporting Information Available

Free-energy values extracted from ED analysis of [Au_25_(GSH)_18_]^−1^ nanocluster, analysis of intermolecular H-bonds of 13 [Au_25_(GSH)_18_]^−1^-(H_2_O)_126_ clusters conformations in relation to individual ECD spectra, B3–1 solvation shell ECD spectrum, Hausdorff chirality measure values for B3–1 conformation, additional analysis of selected solvation shell, ECD spectra calculated with different xc functionals of the most probable conformer B3–1 (PDF)
