# Supplementary material for: What Contributes to the Measured Chiral Optical Response of the Glutathione-Protected Au25 Nanocluster?
Source: ACS Nano. 2023 Jun 8;17(12):11481–91. doi: 10.1021/acsnano.3c01309 (PMC10311591; doi:10.1021/acsnano.3c01309)
Supplement: Supplementary file 1 — nn3c01309_si_001.pdf [file nn3c01309_si_001.pdf]

Supporting Information for:

What Contributes to the Measured Chiral Optical Response of the Glutathione-Protected Au_{25} Nanocluster?

Marta Monti[1], María Francisca Matus[2], Sami Malola[2], Alessandro Fortunelli[3], Massimiliano Aschi[4], Mauro Stener[1,a,*], Hannu Häkkinen[2,5,b,*]

[1] Dipartimento di Scienze Chimiche e Farmaceutiche, Università di Trieste, Via L. Giorgieri 1, 34127 Trieste, Italy.

[2] Department of Physics, Nanoscience Centre, University of Jyväskylä, P. O. Box 35, FI-40014, Finland.

[3] CNR-ICCOM, Consiglio Nazionale delle Ricerche, via G. Moruzzi 1, 56124, Pisa, Italy.

[4] Dipartimento di Scienze Fisiche e Chimiche, Università dell'Aquila, Via Vetoio, 67100, l'Aquila, Italy.

[5] Department of Chemistry, Nanoscience Centre, University of Jyväskylä, P. O. Box 35, FI-40014, Finland.

[a]Email: stener@units.it

[b]Email: hannu.j.hakkinen@jyu.fi

Table S1. Projections and ΔG° values of the 7 $[Au_{25}(GSH)_{18}]^{-1}$ conformations extracted from the ED analysis of the full-MD simulation.

$[Au_{25}(GSH)_{18}]^{-1}$ Conformation	$\Delta G^{\circ}_{[Au_{25}(GSH)_{18}]^{-1}}$ (kJ/mol)
B1	0.87
B2	1.45
B3	0.00
B4	2.21
B5	0.34
B6-A	2.03
B6-B	1.40

Relation between inter-molecular H-bonds and ECD features

We have investigated the correlation of the ECD spectra of the 13 $[Au_{25}(GSH)_{18}]^{-1}$-$(H_2O)_{126}$ clusters and the number of H-bonds that each conformation establishes with its corresponding solvent shell. All the results are collected in Figure S1 which confirms how strong the solvent effect is on the nanocluster, as already discussed in this and previous works[Error! Bookmark not defined.]. For instance, we can notice an almost opposite phase between 4.5-5 eV for the B2 (2th row panels), and B4 ECD spectra (4th row panels), even though there is only a difference in the solvation shell conformation. Instead, for the B1 clusters (1st row panels) which have *ca.* the same number of H-bonds, no differences are observed in the spectral features. The B3 conformations (3rd row panels) show a similar number of H-bonds as well, but some differences can be noticed from 4.5 eV onward, mostly due to a blueshift of the B3-2 ECD spectrum. It is worth noting that the average H-bonds value for the B3 constrained-MD simulation is the lowest one (*i.e.,* 117), while they oscillate between 127 (B6-A) and 141 (B2) bonds for the other simulations. This result supports what was found in Section 3.1 (see Figure 4c), *i.e.,* the B3 nanocluster conformation exhibits a higher number of intra-molecular H-bonds with respect to the other selected low-energy conformations. These analyses together with the two B3 ECD spectra suggest that conformations with a larger number of intra-molecular H-bonds produce a better agreement with the experimental spectrum in the high-energy range (maximum peak). The hypothesis is supported also by B2, B4 and B5 conformations where the conformation with fewer inter-molecular H-bonds gives the maximum feature, while the trend is refuted by those extracted from B6-B which show a strong decreasing behavior in this high energy region.

Furthermore, we observed the overlap between the two ECD spectra (lowest panel of Figure S1) even though the solvation shells of B6-B – 1 and – 2 are very different in terms of nanocluster–water interactions. For B6-A – 1 instead, we obtained the expected maximum peak between 4.5-5 eV, although significantly reduced if we consider that the number of inter-molecular H-bonds is quite small (similar to the B3 case). However, we already observed that the nanocluster conformations from B6 deviate from the H-bond trend found for the other low-energy conformations, and similar discrepancies arise now analyzing the inter-molecular interactions.

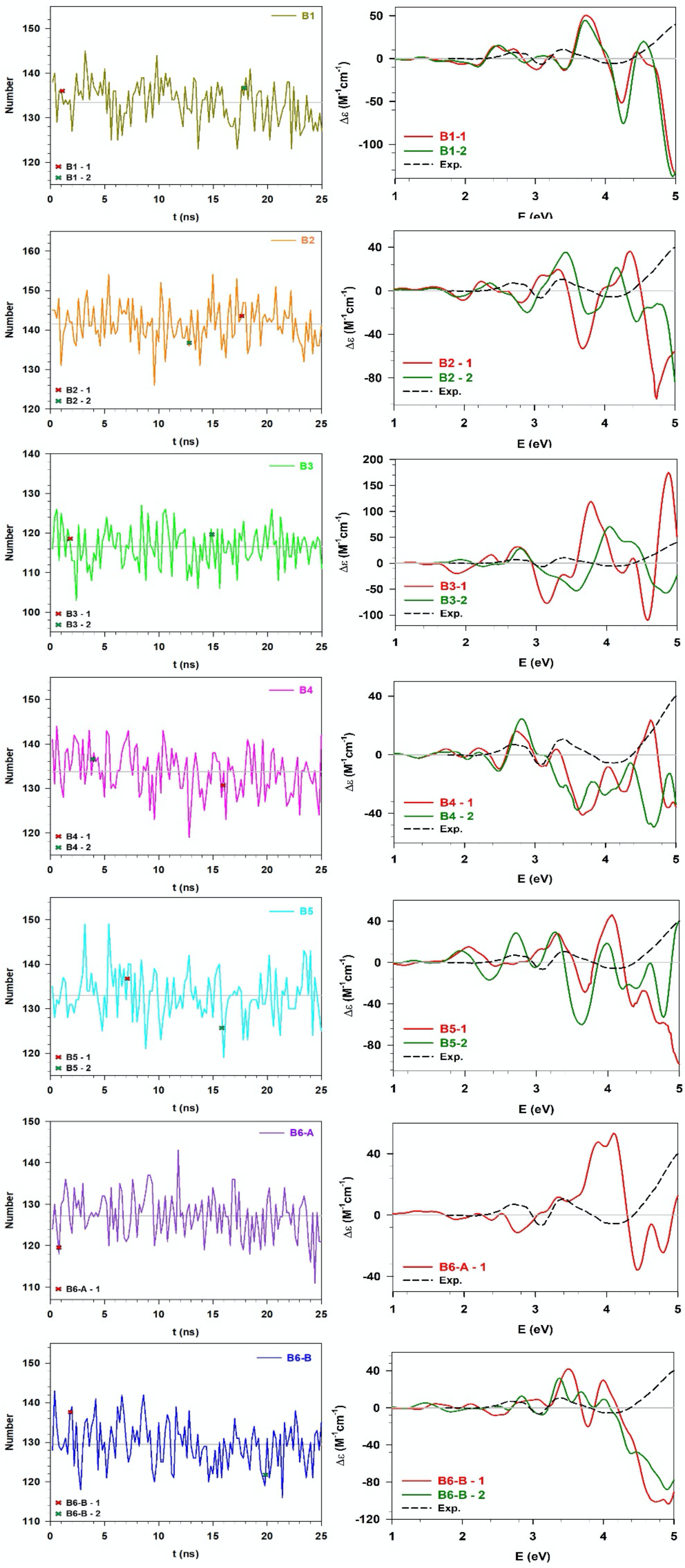
B1
B1 - 1
B1 - 2
B1-1
B1-2
Exp.
B2
B2 - 1
B2 - 2
Exp.
B3
B3 - 1
B3 - 2
B3-1
B3-2
Exp.
B4
B4 - 1
B4 - 2
Exp.
B5
B5 - 1
B5 - 2
B5-1
B5-2
Exp.
B6-A
B6-A - 1
Exp.
B6-B
B6-B - 1
B6-B - 2
Exp.
Number
t (ns)
Δε (M⁻¹cm⁻¹)
E (eV)

Figure S1. Left panels: inter-molecular H-bonds (a.u.) of $[Au_{25}(GSH)_{18}]^{-1}$-$(H_2O)_{126}$ clusters along the 25 ns constrained-MD simulations. The clusters conformations extracted (see Table 2) from each constrained-MD are reported with colored crosses in correspondence of their time step in the simulation. The average inter-molecular H-bonds values (<H-bonds>) are reported in the upper left region of each spectrum. Right panels: individual (not weighted) ECD spectra of the 13 conformations.

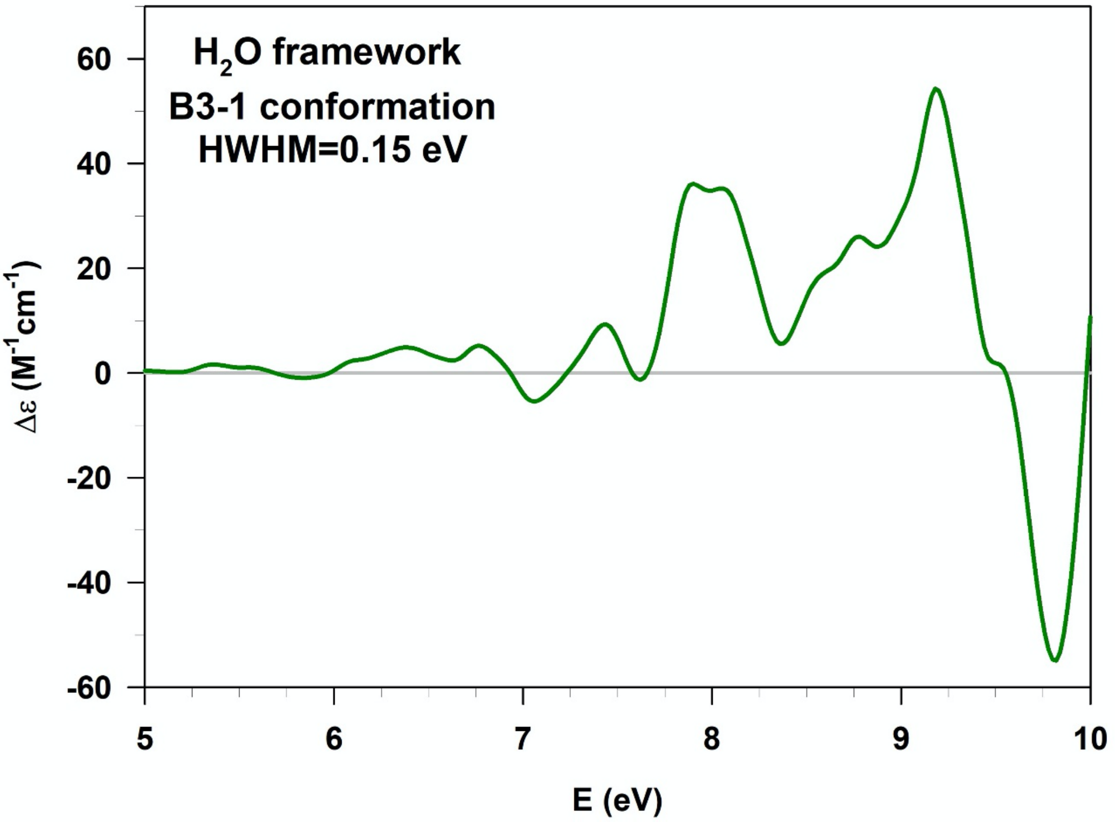

Figure S2. Calculated ECD for the solvation shell of the most probable conformer extracted in this work (B3-1, see Table 1). The spectrum has been calculated employing the LB94 xc functional and a Half Width Half Maximum (HWHM) equals to 0.15 eV.

Table S2. Hausdorff chirality measure (HCM) [1,2] calculated for estimating the chirality of the most probable conformer extracted in this work (B3-1, see Table 1). The HCM values of $[Au_{25}(GSH)_{18}]^{-1}$, the solvation shell ($(H_2O)_{126}$), and the whole system ($[Au25(GSH)_{18}]^{-1}(H_2O)_{126}$) are reported separately.

$[Au_{25}(GSH)_{18}]^{-1}$	$(H_2O)_{126}$	$[Au_{25}(GSH)_{18}]^{-1}(H_2O)_{126}$
0.127	0.127	0.0868

Additional details on the selected solvation shell

The glutathione (GSH) ligand shown in Figure S3 presents several sites that can directly interact with the surrounding water molecules. For instance, -CO, -NH, $-NH_3^+$, and $-CO_2^-$ groups can be involved in H-bonds or charge-dipole interactions with the solvent.

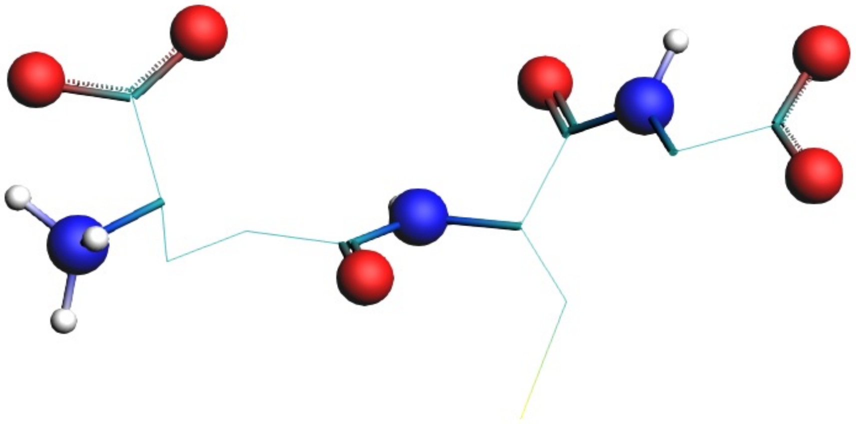

Figure S3. Individual glutathione (GSH) ligand. -CO, -NH, $-NH_3^+$, and $-CO_2^-$ groups are represented as sticks and balls (red for O, cyan for C, blue for N, and white for H), while the remaining C, and S atoms are shown with a wireframe model (H atoms are not shown).

Therefore, we can estimate an average of 9 sites per ligand for possible inter-molecular interactions with the water molecules. Since 18 GSH ligands compose the nanocluster, we must take into account that certain sites will be involved in intra-molecular interactions or will be inaccessible for steric effects. For simplicity, we only considered the first possibility analyzing the full-MD fluctuations of the intra-molecular H-bonds (see Figure 4c) whose average value is around 32, thus an approximatively average of 2 sites per ligand involved in these interactions. Starting from these considerations, we extracted solvation shells of 7x18 water molecules which qualitatively represent the physics of the aqueous system, thus can be used to mimic the real effects of the water solvent on the nanocluster. As we can notice from Figure 1, a qualitative good solvation of the whole ligands is obtained with the water molecules located both in the inner and outer parts of the nanocluster.

We also briefly analyzed the solvation in terms of radial distribution function (g(r)) of the water molecules with respect to the core gold atom (AuC) to estimate the number of shells that must be included for a proper solvation of this system. The results are shown in Figure S4.

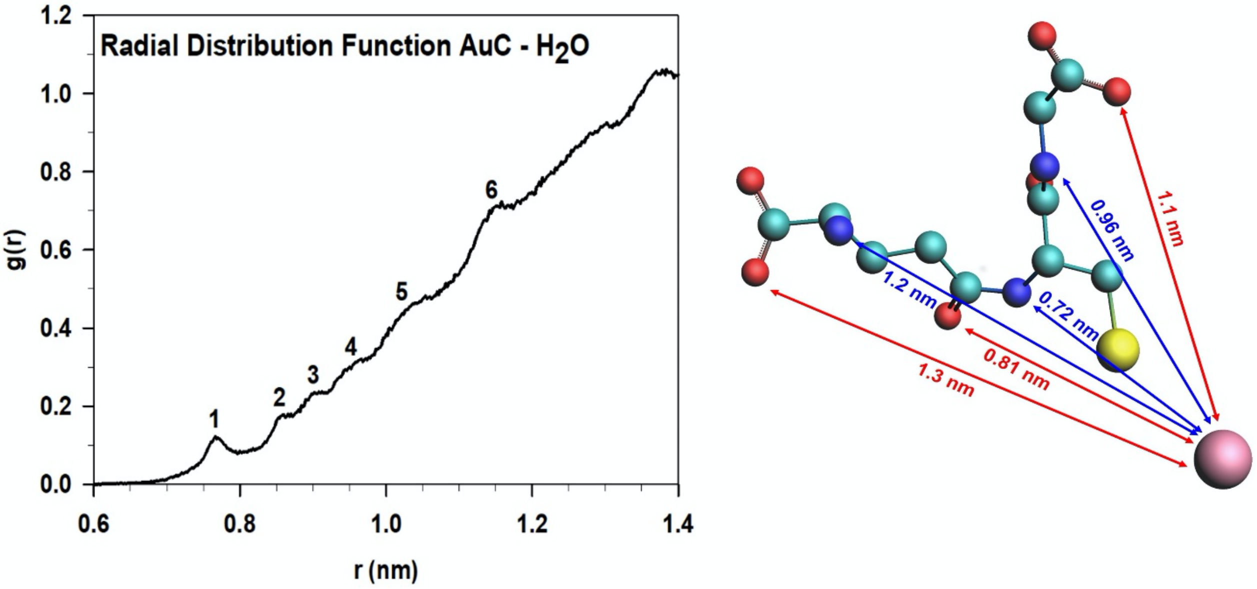

Figure S4. Radial distribution function g(r) of the water molecules with respect to the core gold atom AuC on the left panel. The first 6 solvation shells are highlighted in the figure. Example of the distances of the GSH sites, which can directly interact with the water molecules, with respect to the AuC atom on the right panel. All the distances are reported in nm.

We investigated the g(r) behavior together with the approximate distances of the ligand regions from the core gold atom AuC to evaluate which is the range in nm that should be covered for the solvation. Estimating a distance of ~ 1.2/3 nm with respect to the AuC, at least 6 shells of solvation in terms of g(r) should be considered. It is worthy of note that it is quite common to use only the first shell in order to simulate the solvation of a certain system, which in this case would be a much stronger approximation with respect to the one proposed in this work. Indeed, in that case only the inner part of the ligand would be solvated leaving the chiral C atom of the glutamate residue, which is external, less exposed to the water molecules.

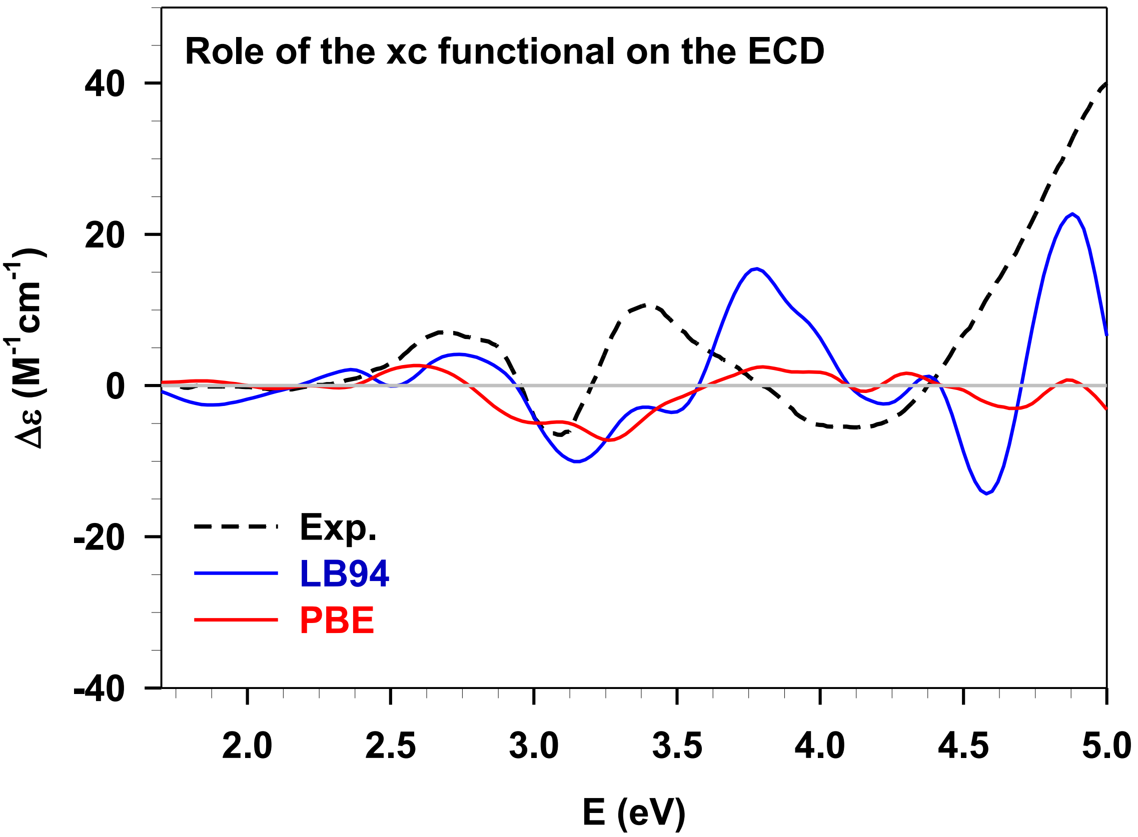

Figure S5. Experimental (Exp., black dashed line) ECD of the solvated $[Au_{25}(GSH)_{18}]^{-1}$ and the ECD calculated for the most probable conformation (B3-1, see Table 2 in the Main Article) employing the LB94 (solid blue line) and PBE (solid red line) xc functionals. The calculated ECD have been multiplied by the statistical weight of this conformation (0.13, see Table 1).

---

[1] Buda, A. B.; Mislow, K. A Hausdorff Chirality Measure. *J. Am. Chem. Soc.* **1992**, *114*, 6006-6012.

[2] Garzón, I. L.; Reyes-Nava, J. A.; Rodríguez-Hernández, J. I.; Sigal, I.; Beltrán, M. R.; Michaelian, K. Chirality in Bare and Passivated Gold Nanoclusters. *Phys. Rev. B* **2002**, *66*, 073403.
